# A Causal Relationship between Vitamin C Intake with Hyperglycemia and Metabolic Syndrome Risk: A Two-Sample Mendelian Randomization Study

**DOI:** 10.3390/antiox11050857

**Published:** 2022-04-27

**Authors:** Meiling Liu, Sunmin Park

**Affiliations:** Department of Food and Nutrition, Obesity/Diabetes Research Center, Hoseo University, Asan 31499, Korea; 20185752@vision.hoseo.edu

**Keywords:** vitamin C, metabolic syndrome, hyperglycemia, hypertension, type 2 diabetes, Mendelian randomization

## Abstract

Excessive oxidative stress can contribute to metabolic syndrome (MetS), and antioxidants can protect against its development. Vitamin C (VC) is a well-known antioxidant, and observational studies have associated a deficiency with an increased MetS risk. This study tested the hypothesis that dietary VC intake caused an inverse relation of MetS and its components risk using a two-sample Mendelian randomization (MR) method in adults ≥40 years in a city hospital-based (*n* = 58,701) and Ansan/Ansung plus rural (*n* = 13,598) cohorts. Independent genetic variants associated with dietary VC intake were explored using a genome-wide association study (GWAS) with significance levels of *p* < 5 × 10^−5^ and linkage disequilibrium (r2 threshold of 0.001), after adjusting for the covariates related to MetS, in a city hospital-based cohort (*n* = 52,676) excluding the participants having vitamin supplementation. MR methods, including inverse-variance weighting (IVW), weighted median, MR-Egger, and weighted model, were used to determine the causal relationship between the dietary VC intake and the risk of MetS and its components in Ansan/Ansung plus rural cohorts (*n* = 11,733). Heterogeneity and leave-one-out sensitivity analyses were conducted. Energy intake, as well as other nutrient intakes, were significantly lower in the low VC intake group than in the high VC intake group, but the incidence of MetS and its components, including hyperglycemia, hypertriglyceridemia, and hypertension, was observationally higher in inadequate low VC intake in the combined cohorts. In MR analysis, insufficient dietary VC intake increased the risk of MetS, hyperglycemia, hypertriglyceridemia, and hypertension in an IVW (*p* < 0.05). In contrast, only the serum fasting blood glucose concentration was significantly associated with VC intake in weight median analysis (*p* < 0.05), but there was no significant association of low dietary VC with MetS and its components in MR-Egger. There was no likelihood of heterogeneity and horizontal pleiotropy in MetS and its components. A single genetic variant did not affect their association in the leave-one-out sensitivity analysis. In conclusion, insufficient dietary VC intake potentially increased the MetS and hyperglycemia risk in Asian adults. Low VC intake can contribute to increasing type 2 diabetes incidence in Asians.

## 1. Introduction

Metabolic syndrome (MetS) is a cluster of abdominal obesity, dyslipidemia, hyperglycemia, and hypertension that can progress to metabolic diseases, type 2 diabetes, and cardiovascular diseases [1]. The common etiology of MetS is insulin resistance developed when insulin action is attenuated due to aging, obesity, sedentary lifestyles, smoking, and sleep apnea [2,3]. When insulin resistance increases, elevated insulin secretion overcomes insulin resistance to normalize the serum glucose concentrations [3]. Under insulin resistant circumstances, reactive oxygen species (ROS) and proinflammatory cytokines are elevated to activate c-Jun *n*-terminal kinases (JNK1), nuclear factor kappa-light-chain-enhancer of activated B cells (NF-kB), and mitogen-activated protein kinase (MAPK) [4]. Therefore, insulin resistance, chronic inflammation, ROS, and oxidative stress make a vicious cycle, leading to metabolic diseases, and antioxidants can improve MetS risk [5,6,7].

Oxidative stress develops when the antioxidant system does not eliminate excessive oxidative products [7]. Excessive oxidative products disrupt proteins, lipids, and nucleic acids, resulting in cellular metabolism dysfunction, including insulin resistance [8]. Both high fat and high carbohydrate diets are reported to elevate oxidative stress because of increased protein carbonylation and lipid peroxidation products and decreased antioxidant system, the glutathione (GSH) levels, and antioxidant enzymes, including superoxide dismutase (SOD), GSH peroxidase, and catalase [7]. Adults with MetS have suboptimal concentrations of retinyl esters, vitamin C (VC), and carotenoids [9], and may be associated with insufficient intake of antioxidants. 

VC is involved in collagen production, which helps maintain vascular aging [10]. VC intake measured by the food frequency questionnaire has a moderate relationship with plasma VC concentration in meta-analysis [11]. VC intake can be used as a biomarker of VC status. VC deficiency contributes to vascular stiffness related to fibrosis, perivascular inflammation, and vascular calcification [10]. In observational studies, dietary VC and fruit intake are inversely associated with the MetS risk and help maintain VC concentration in systemic circulation [12,13]. Among the MetS components, inadequate VC intake is positively related to type 2 diabetes risk [14]. Hyperglycemia elevates ROS production by glucose autooxidation to exacerbate the diabetic condition by increasing beta-cell apoptosis, contributing to deteriorating β-cell function and insulin sensitivity [15,16]. ROS directly exacerbates insulin resistance. Hyperglycemia also increases advanced glycated end products by non-enzymatic reaction contributing to diabetic complications, while high VC intake reduces fasting serum glucose and HbA1c concentrations in type 2 diabetic patients [15]. Furthermore, obesity and fatty liver disease contribute to reducing the plasma VC concentration, and body fat contents are inversely associated with plasma VC concentration linked to dietary VC intake [15]. On the other hand, no causal relationship between the dietary VC intake and MetS and its components has been conducted.

In observational studies, Mendelian randomization (MR) uses the measured genetic variation to examine the causal effect of a modifiable exposure on disease as people are born with random genetic variants [17]. MR represents the casual relationships of modifiable variables in disease development because it is minimally influenced by clinical confounders or reverse causation [17]. We hypothesized that dietary VC intake was causally and inversely associated with MetS and its components. We examined the hypothesis by a two-sample MR study in 64,391 adults aged ≥ 40 years who belonged to two different cohorts in a Korean Genome Epidemiology Study (KoGES). The present study was the first to show a causal relationship between the VC intake from foods and the risk of MetS and its metabolic traits in Asian adults.

## 2. Materials and Methods

### 2.1. Participants

During 2004–2013, 71,568 middle-aged and elderly Korean adults were recruited voluntarily in the KoGES organized by the Korean Center for Disease and Control (*n* = 72,299). The Ansan/Ansung (*n* = 5493), rural-based (*n* = 8105), and city hospital-based cohorts (*n* = 58,701) in KoGES were combined for the present study. Since the vitamin supplements did not include their product information in KoGES data, the VC amounts to be taken were unknown. It is unclear to which the participants taking vitamin supplementation should belong. Therefore, the participants with any vitamin supplementation were excluded (*n* = 7908). Meanwhile, we also conducted MR analysis in the KoGES data, including the participants having vitamin supplementation: they were divided into low and high VC groups according to dietary VC intake, and vitamin supplementation was included as a covariate. The data including and excluding vitamin supplementation showed similar results in the effects of VC intake on MetS and its components. We excluded the participants taking multivitamin supplementation to eliminate the uncertainty of VC inclusion in the multivitamin supplementation: 52,658 participants in a city hospital-based cohort and 11,733 ones in the Ansan/Ansung plus rural cohorts. The present study followed the Helsinki Declaration and was approved by the Institutional Review Board on KoGES (KBP-2019-055) and the Institutional Review Board of Hoseo University (1041231-150811-HR-034-01). All subjects who participated in KoGES provided written informed consent.

### 2.2. Clinical Parameters Measurements 

The participants had a health interview and survey, including age, education, income, smoking status, alcohol intake, and physical activity [18]. Education was classified into three groups: under high school, high school, and college or more. Household income was categorized into four groups (USD/month): very low (<1000), low (1000–2000), inter-mediate (2000–4000), and high (>4000) [19]. The smoking status was divided into a current smoker, past smoker, and never-smoker, according to smoking >100 cigarettes within six months [20]. The daily alcohol intake was estimated with the alcohol types, amounts, and frequencies, and categorized into nondrinker, light drinker (<20 g), and moderate drinker (>20 g) [19]. The regular intake of multivitamin complexes was reported.

Anthropometry and biochemical measurements were conducted. The body weight, height, and waist circumference were measured using a standardized procedure [21]. The body mass index (BMI) was calculated by dividing the body weight (kg) by the square of the height (m^2^). Blood from the participants without food for more than 12 h was collected with and without anti-coagulators. The plasma and serum were separated from the collected blood after centrifugation. The glucose, aspartate aminotransferase (AST), alanine aminotransferase (ALT), creatinine, and lipid profiles were measured from the plasma and serum samples using an Automatic Analyzer (Hitachi 7600, Hitachi, Tokyo, Japan) [21]. Serum high–sensitive C–reactive protein (hs–CRP) concentrations were assessed with an ELISA kit. Hemoglobin A1c was determined from the blood. The SBP and DBP on the right arm were measured three times at the same height as the heart in the sitting position, and the average values were used.

### 2.3. MetS Definition 

According to the modified 2005 revised National Cholesterol Education Pro-gram-Adult Treatment Panel III criteria for Asia, the participants were considered to have MetS if they met three or more of the following criteria [17,18]: (1) abdominal obesity (waist circumferences ≥ 90 cm for men and ≥85 cm for women; (2) elevated blood pressure (aver-age systolic blood pressure ≥ 140 mmHg or diastolic blood pressure ≥ 90 mmHg) or taking current blood pressure medication; (3) elevated fasting blood glucose concentration (≥100 mg/dL) or current use of anti-diabetic medication; (4) low HDL-C concentration (<40 mg/dL for men and <50 mg/dL for women) or taking anti-dyslipidemic medication; (5) elevated serum triglyceride concentration (≥150 mg/dL) or taking anti-dyslipidemic medication.

### 2.4. Food and Nutrient Intake 

The food intakes were assessed using a semi-quantitative food frequency questionnaire (SQFFQ) designed and developed for the Korean eating patterns and validated during the KoGES [22]. The usual food intake was asked over the previous six months, and the questionnaire requested information regarding the intake of 106 food items. From the SQFFQ information, 23 nutrient intakes were calculated using the Computer-Aided Nutritional Analysis Program (CAN Pro) 3.0, a nutrient database program established by the Korean Nutrition Society [22].

### 2.5. Dietary Inflammatory Index (DII) 

DII, an index of the dietary inflammatory index, was calculated from the equation with assigned food and nutrient intakes using their dietary inflammatory weights, including the energy, 32 nutrients, four food products, four spices, and caffeine, as described previously [23]. Because the SQFFQ did not include garlic, ginger, saffron, and turmeric, their intake was excluded from the DII calculation. DII was calculated by multiplying the dietary inflammatory scores of the 38 food and nutrient components by the daily intakes, and the sum of the scores of 38 items was divided by 100.

### 2.6. Genotyping

The genomic DNA was extracted from the whole blood of the participants in the hospital-based cohort study. Any participants were not in the same family. The genotypes were measured on a Korean Chip (Affymetrix, Santa Clara, CA, USA), designed to study Korean genetic variants involved in diseases prevalent in Koreans [24]. The genotyping accuracy was examined by Bayesian robust linear modeling using the Mahalanobis distance (BRLMM) genotyping algorithm [25]. The Biobank that belongs to the Korea National Institute of Health was organized to collect the data from the cohort and has provided genotype data for research. DNA samples had quality control to include no gender biases, genotyping accuracies (≥98%), low heterozygosity (<30%), missing genotype call rates (<4%), minor allele frequency (MAF, >1%), and HWE (*p* > 0.05) [25,26].

### 2.7. Two-Sample MR Analysis Design

**Figure 1:**
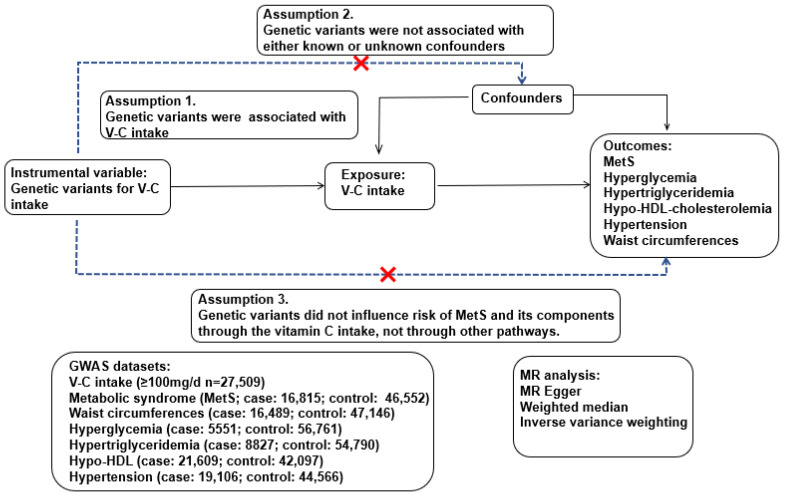
Experimental design for the Mendelian randomization (MR) analysis of the vitamin C (VC) intake with the metabolic syndrome risk.

The genetic variants associated with the dietary VC intake (interested exposure) were used as an instrumental variable in a two-sample MR analysis (Figure 1). If the dietary VC intake (exposure) causes MetS and its components (outcomes), the genetic variants affecting the VC intake (exposure) should be associated with MetS and its components (outcome). This MR analysis assumed the following: (1) the genetic variants were associated with VC intake; (2) the genetic variants related to VC intake were not associated with confounders; (3) the genetic variants related to VC intake did not influence the risk of MetS and its components [27]. The confounders included continuous variables (age, BMI, and energy and alcohol intake) and categorical variables (residential area, gender, education, income, exercise, and smoking status). These assumptions were checked at a statistical significance level of *p* < 5 × 10^−5^, and these assumptions were satisfied in the present study. Therefore, VC intake was causally associated with MetS risk when MR analysis between dietary VC intake and MetS and its components showed a significant relation.

The Mendelian randomization package (version 0.5.1) and the two-sample MR (version 0.4.26) in the R program were used for the two-sample MR analysis. A two-sample MR study was applied to determine the causal association of dietary VC intake (exposure) with MetS and its components (outcomes) using genetic variants to influence dietary VC intake. We used two different cohorts in KoGES to satisfy a two-sample MR assumption: an instrumental variable was generated from the city hospital-based cohort (*n* = 52,676; low VC: *n* = 29,398; high VC: *n* = 22,729) while the association of an instrumental variable with MetS and its components was conducted in the Ansan/Ansung plus rural cohorts (*n* = 11,733) using the MR package, including in the IVW method, MR-Egger, weighted median, and weighted mode. The number of participants in MetS and its metabolic traits in the Ansan/Ansung plus rural cohorts, are as follows: (1) MetS (cases, *n* = 4279; control, *n* = 7435); (2) hypertension (cases, *n* = 4355; control, *n* = 7366); (3) fasting serum glucose concentration (cases, *n* = 999; control, *n* = 10,712); (4) waist circumferences (cases, *n* = 4144; control, *n* = 7572); (5) serum HDL-C concentration (cases, *n* = 6277; control, *n* = 5455); (6) serum triglyceride concentration (cases, *n* = 2306; control, *n* = 9424). 

The Wald ratios were conducted for each SNP using the IVW method. When the data points were constrained into the intercept zero in the plot of outcome and exposure, the gradient of the line gave a strong association. The slope indicates how much a unit increases in the outcome relative to each increment unit of VC intake (exposure) [27]. Horizontal pleiotropy may induce bias in the IVW method.

The MR-Egger method consists of (1) testing for directional pleiotropic effects, (2) examining a causal effect, and (3) estimating a causal effect. The directional pleiotropic effects by MR-Egger indicate that the selected SNPs can affect the outcome through direct actions on a specific pathway other than exposure variables. The line does not necessarily have to pass through zero [28], and the intercept estimates the directional pleiotropic effect. The MR-Egger method has the lowest power among MR methods in identifying a causal relationship between exposure and outcome [29]. It is more effective when more SNPs are included as the instrument variables. No measurement error exists in the SNP and exposure effects in the MR-Egger method. 

The weighted median approach was determined under the assumption that more than 50% of the total weight of the instrument variables comes from valid genetic variants. The weight mode method estimates a stronger causal relationship than the MR-Egger or IVW [28,29]. 

### 2.8. Instrumental Variable in MR Analysis: Genetic Variants for VC Intake 

Dietary VC intake was categorized with a cutoff of 100 mg/day, a recommended intake in dietary reference intake [30]: low VC group (<100 mg VC/day intake), and high VC group (≥100 mg VC/day intake). A genome-wide association study (GWAS) of VC intake was conducted with low VC and high VC groups after adjusting for the mentioned confounders using PLINK version 2.0 (http://pngu.mgh.harvard.edu/~purcell/plink; accessed on 22 July 2021) in the city hospital-based cohort (*n* = 52,676). The Manhattan plots and quantile–quantile (Q–Q) plots were generated using the “fastman” and “data.table” library package in the R program. 

The significance of genetic variants in GWAS for diseases was used with *p* < 5 × 10^−8^, but in the GWAS of VC intake, no higher SNPs were obtained than *p* < 5 ×10^−7^. Meanwhile, lifestyle parameters have not shown high significance with genetic variants in previous studies, and a liberal *p*-value threshold of 5 × 10^−5^ is applied when using lifestyles as the instrument variables [28,29,31]. Additionally, the application of Bonferroni correction might be too conservative since many SNPs having linkage disequilibrium (LD) relation were not independent [32]. Therefore, a *p*-value threshold of 5 × 10^−5^ was applied for the VC intake in the present study. Among those highly associated with VC intake from GWAS (*p* < 5 × 10^−5^), genetic variants with LD pruning (distance threshold = 10,000 kb; r2 threshold of 0.001) were excluded using the clump_data command in the TwoSampleMR package in R program [33]. The r^2^ threshold of 0.001 is equivalent to D′ < 0.2 in LD analysis in Plink [33]. An independent set of 42 genetic variants was selected as an instrumental variable for VC intake from the city hospital-based cohort. Single nucleotide polymorphism (SNP)-associated genes were identified by searching the g:profiler database site (https://biit.cs.ut.ee/gprofiler/snpense; accessed on 30 July 2021).

### 2.9. Statistical Analysis 

Statistical analysis was conducted using SAS version 9.3 (SAS Institute, Cary, NC, USA). Descriptive statistics of the categorical variables (e.g., gender, education, and smoking status) were analyzed using the frequency distributions in the low VC and high VC intake groups. The significant differences between their frequency distributions were assessed using a chi-squared test. According to the V-C intake, the means and standard deviations were expressed in the continuous variables. The significance of the differences among the VC intake groups was analyzed using an analysis of covariance (ANCOVA) with adjusting covariates. The association of demographic, lifestyles, and nutrient intake, including VC, with MetS risk, was analyzed using logistic regression analysis for categorical variants and linear regression for continuous variables with an adjustment for covariates in the observational study. The adjusted odds ratios (ORs) and 95% confidence intervals (CI) were observationally and causally calculated for the MetS risk against high VC intake (≥100 mg/day) as the reference. Covariates included adjustment for age, sex, residential area, education, income, BMI, smoking status, alcohol consumption status, and overall physical activity and energy intake.

## 3. Results

### 3.1. Clinical Characteristics

Age was higher in the low VC group than in the high VC group, and age was negatively associated with the VC intake (Table 1). More men in the low VC group than women and men were inversely associated with VC intake by 0.563 times. The BMI was slightly but significantly higher in the high VC group than in the low VC group, but the waist circumferences were opposite of the BMI. The BMI was positively associated, but waist circumferences were negatively associated with the dietary VC intake in linear regression analysis. People with high education and income had a higher VC intake than those with low education and income. Education and income were positively associated with the VC intake (Table 1). On the other hand, more participants were currently smoking and consuming alcohol in the low VC group than in the high VC group. There was an inverse association between current smokers and alcohol intake and dietary VC intake. Physical exercise showed an opposite trend to smoking status in the dietary VC intake (Table 1). These results suggest that sufficient dietary VC may be linked to self-health consciousness.

The participants with a high VC intake had a lower incidence of MetS than those with a low VC intake. The high VC group was inversely associated with the MetS risk. In the MetS components, the fasting serum glucose concentrations and HbA1c concentrations, diabetic indices, were negatively associated with the VC intake in linear regression (Table 1). On the other hand, serum total cholesterol and LDL concentrations were not significantly different, and serum HDL concentrations were positively associated with VC intake. Serum triglyceride concentrations were negatively associated with the VC intake. SBP, but not DBP, exhibited a negative association with the VC intake in linear regression (Table 1). The serum hs–CRP concentrations, an inflammation index, were higher in the low VC group than in the high VC group and negatively correlated with VC intake. Blood hemoglobin and hematocrit contents were positively associated with VC intake, which may promote Fe intake by VC intake (Table 1). The estimated glomerular filtration rate was positively associated with the VC intake (Table 1). There were no significant differences in the serum AST and ALT concentrations (liver damage indices) between the low VC and high VC groups (Table 1).

### 3.2. Nutrient Intake

The VC intake of the high VC group was approximately 2.6 times higher than that of the low VC group (Table 2). Interestingly, the daily energy intake based on the estimated energy requirement (EER) was much higher in the high VC group (113.2 EER%) than in the low VC group (86.7 EER%) (Table 2). The low VC group participants consumed higher carbohydrate intake and lower protein and fat intake than those in the high VC group. Saturated, monounsaturated, and polyunsaturated fatty acid consumption was lower in the low VC group than in the high VC group (Table 2). The proportion of saturated, monounsaturated, and polyunsaturated fatty acids was similar in the low VC and high VC groups. On the other hand, cholesterol intake was much higher in the high VC group than in the low VC group (Table 2). The intake of other minerals and vitamins, including Ca, Na, VB1, VD, and fiber, was also much higher in the high VC group than in the low VC group. However, the difference between the low VC and high VC was highest in VC intake than in other nutrients (Table 2). DII, an inflammation index, was also approximately two times higher in the low VC group than in the high VC group (Table 2).

### 3.3. Observational Association of Dietary VC and MetS and Its Components

**Figure 2:**
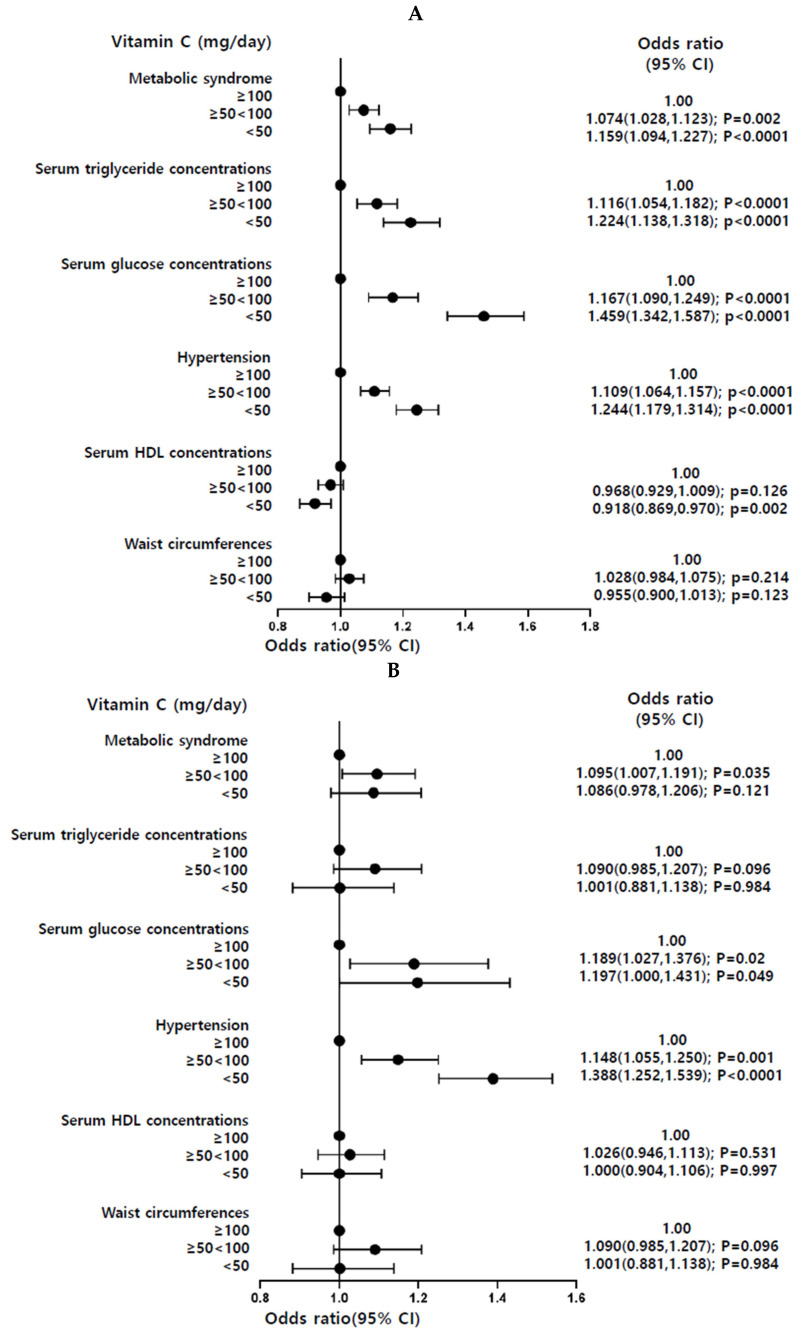
Association of dietary VC intake with the risk of metabolic syndrome (MetS) and its components in observational estimates. (**A**) City hospital-based cohort. (**B**) Ansan/Ansung plus rural cohorts. The reference in the logistic regression was the high VC intake (≥100 mg/day), and it was conducted by adjusting for age, gender, residential area, education, income, BMI, energy intake, exercise, smoking, and alcohol intake.

We separately analyzed the Ansan/Ansung combined with rural (*n* = 11,733) cohorts and the urban hospital-based (*n* = 52,676) cohort. The participants with dietary low VC intake (<100 mg/day) had a higher risk of Mets and its components compared with those with high VC intake (≥100 mg/day; Figure 2). In particular, both cohorts showed that low dietary VC intake significantly increased the risk of hyperglycemia and hypertension (Figure 2).

### 3.4. Causal Association of Dietary VC with MetS and Its Components by a Two-Sample MR Analysis

**Figure 3:**
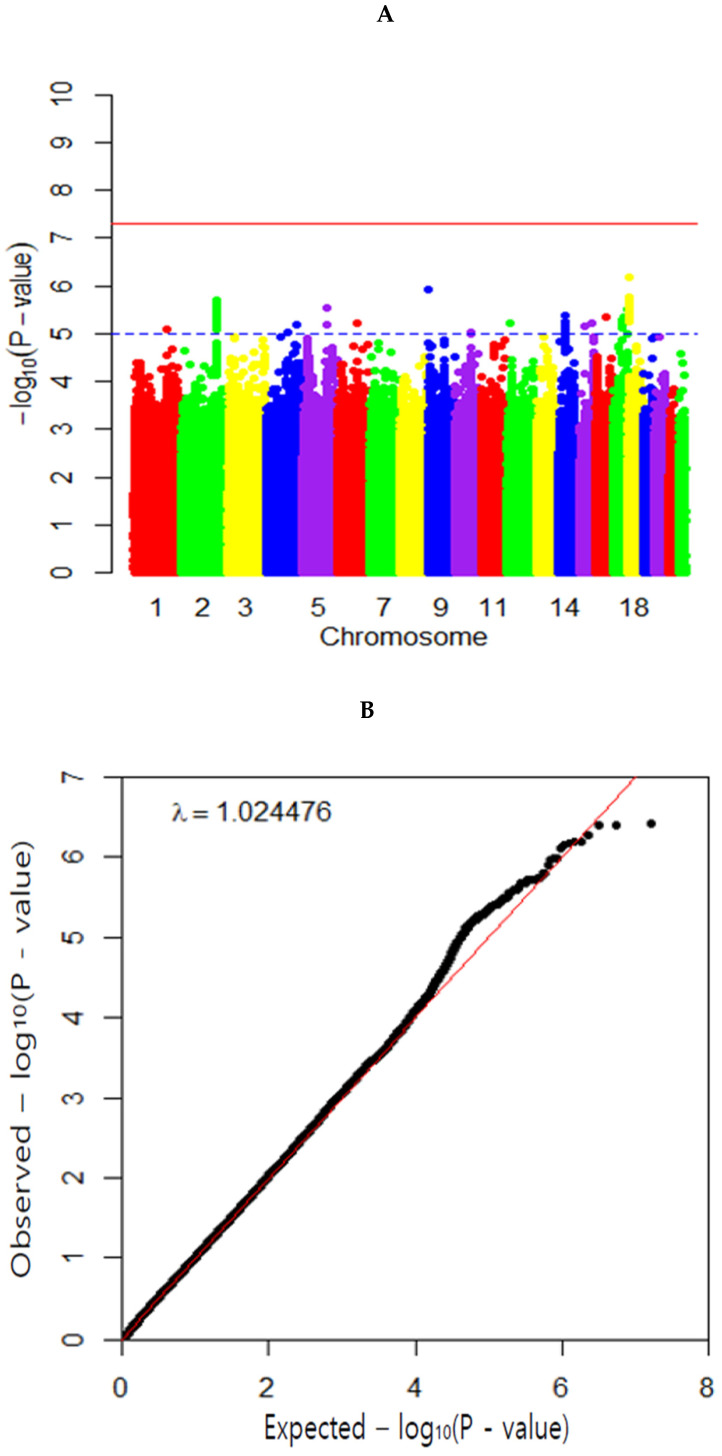
Distribution of genetic variants for dietary vitamin C estimated with genome-wide association study. (**A**) Manhattan plot of the *p*-value of genetic variants for VC intake. (**B**) Q–Q plot of *p*-value based on the association analysis of VC intake.

A causal relationship between dietary VC intake and MetS and its components was determined in the high VC and low VC groups with a cutoff of 100 mg/day, the recommended intake. The Manhattan plot representing *p* values of genetic variants associated with VC intake was shown in Figure 3A. The Q–Q plot shows the quantile distribution of the log of observed *p* values on the y-axis versus the quantile distribution of the log of expected *p* values. The Q–Q plot shows the SNPs from GWAS include no bias to show the association with dietary VC intake. The genome inflation factor was 1.024, close to 1 (Figure 3B). The Q–Q plot and genome inflation factor showed that the *p*-values of SNP had only a few spurious associations for VC intake, indicating the SNPs had no severe bias. For-ty-two genetic variants were selected to satisfy *p* < 5 × 10^−5^ in the GWAS for the VC intake and no potential LD of each other using an r^2^ threshold of <0.001 in the LD analysis within a distance of 10,000 kb. The characteristics of the selected 42 SNPs were provided in Supplemental Table S1. These results indicated that selected genetic variants were associated with VC intake, not correlated with MetS and its components. 

Supplementary Table S1 shows the 42 SNPs used as an instrumental variable for dietary VC intake in a two-sample MR analysis. Low VC intake (<100 mg/day) was causally positively associated with increased risk of fasting serum glucose concentration compared with high VC intake (≥100 from VC intake, IVW β = 0.674, 95% confidence interval (CI) = 0.113–1.235, *p* = 0.018) in the IVW method (Table 3). A genetically-predicted serum glucose concentration was significantly associated with VC intake (Figure 3A). Similar effect estimates were obtained using the weighted median method (β = 0.755, 95% CI = 0.055–1.455, *p* = 0.035). However, MR-Egger did not show a significant VC intake association with serum glucose concentrations (*p* = 0.296) (Table 3), indicating that the selected SNPs for VC intake may be insufficient. The weighted median estimator retained greater precision of the estimates than the MR-Egger analysis, which was often used as a reference for the direction of causal association of the results of the two-sample MR analysis [34]. Therefore, dietary low VC intake showed a causative and positive relationship with serum glucose concentrations. Moreover, low VC intake showed a positive association with MetS and hypertension by 1.49 and 1.55 times in IVM methods, respectively (Table 3). Low VC intake also causally elevated MetS components, including waist circumferences, serum triglyceride, and HDL concentrations in the IVW method. However, dietary VC intake did not significantly correlate with MetS and its metabolic traits in MR-Egger. MR-Egger regression analysis did not suggest evidence of horizontal pleiotropy in MetS and its components (*p* = 0.651–0.985; Table 3). Our study provided evidence that dietary VC intake was causally associated with an increased risk of Mets and its components (Table 3). 

**Figure 4:**
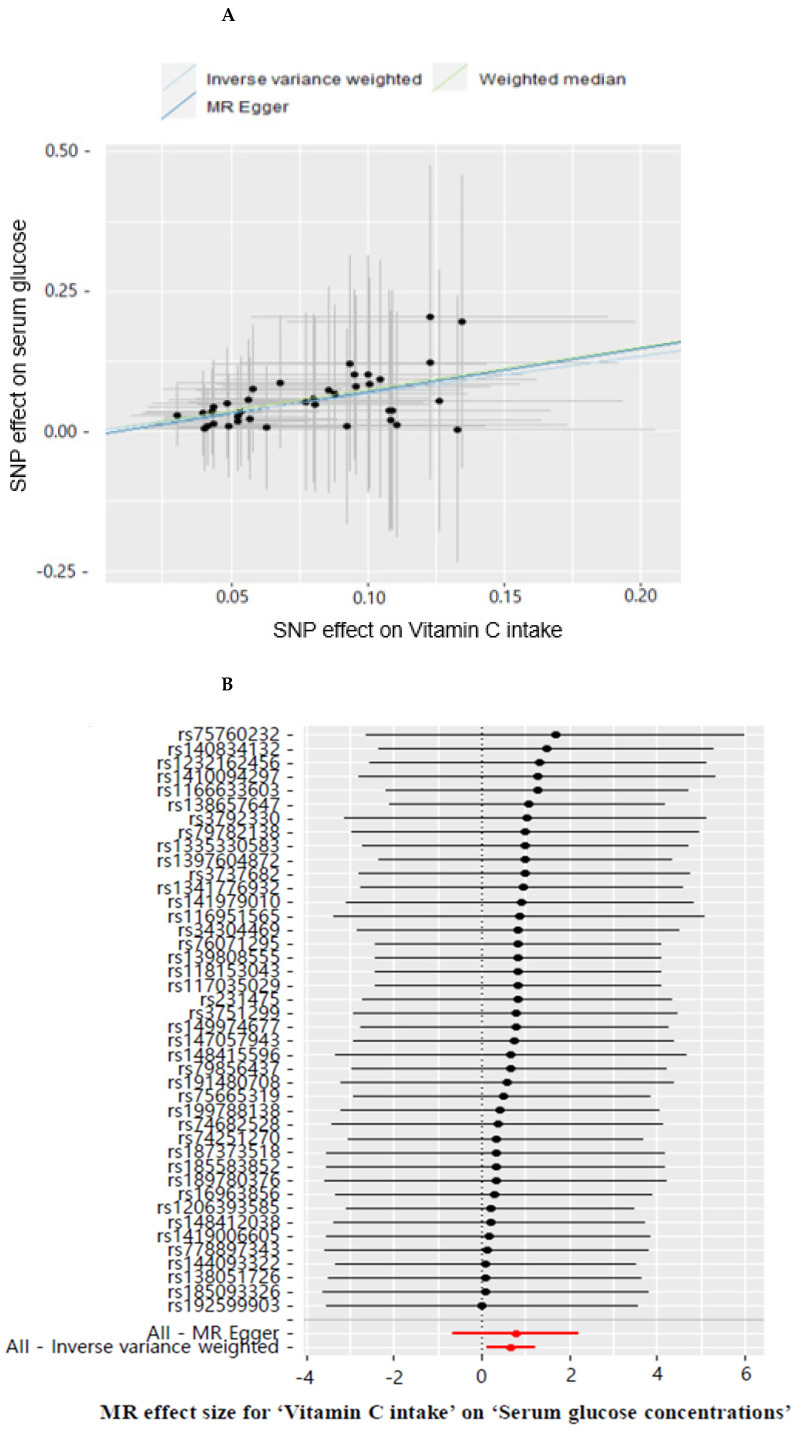

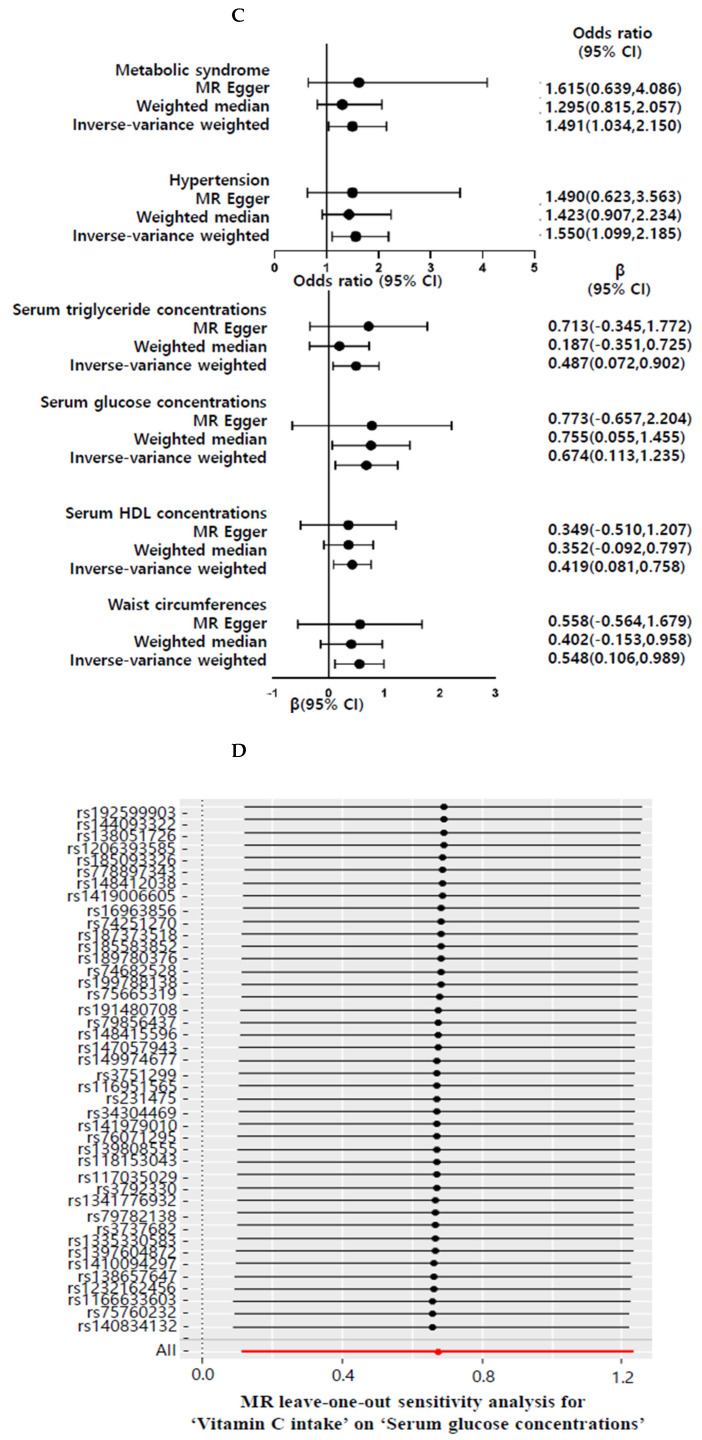

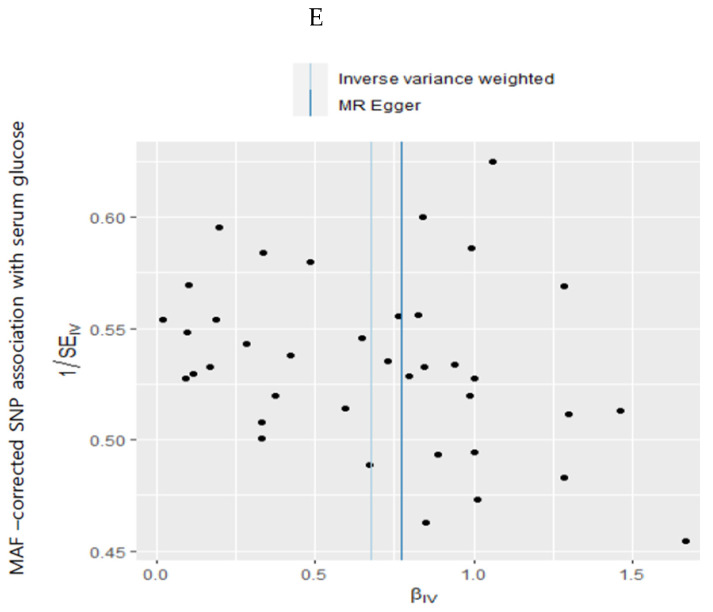
Mendelian randomization (MR) analysis for dietary vitamin C on the serum glucose concentrations. (**A**) Scatter plot of genetic association with the serum glucose concentrations and genetic association with the dietary VC. (**B**) Estimation of MR for the dietary VC and serum glucose concentrations risk. (**C**) Summary of the observational and genetic association of dietary VC with the risk of metabolic syndrome and its components. (**D**) Leave-one-out sensitivity analysis of MR for the dietary VC on the serum glucose concentrations. (**E**) Inverse variance weighted (IVW) and MR-Egger regression funnel plot for the dietary VC on the serum glucose concentrations.

Estimating the heterogeneity based on MR-Egger and IVW methods showed no evidence of heterogeneity in MetS and its components (*p* = 1; Table 3). The horizontal pleiotropy was not significant in the test of the MR-Egger intercepts (*p* > 0.05; Table 3), indicating the low likelihood of horizontal pleiotropy in MetS and its components (Table 3). The leave-one-out method showed that a single genetic variant did not change the association between VC intake and MetS and its components (Figure 4D and Supplemental Figure S1). The MR-Egger regression funnel plot for the serum glucose concentrations showed symmetry in IVW but asymmetry in MR-Egger (Figure 4E). The MR estimate of each SNP was plotted as a function of its MAF-corrected association with the serum glucose concentrations. The MAF correction proportional to the standard error of the SNP–hyperglycemia was used because a low MAF was likely to be measured with low precision. The plot showed symmetry in the IVW and asymmetry in MR-Egger. Therefore, dietary VC intake was causally and inversely associated with hyperglycemia in IVW.

## 4. Discussion

The KNHANES and KoGES showed that inadequate dietary VC intake is positively associated with the MetS risk in Korean adults [2,12], but the results cannot explain the causal relationship based on the cross-sectional data. The present study showed an inadequate VC intake causally elevated the risk of MetS, hypertriglyceridemia, hyperglycemia, and hypertension in a two-sample MR approach (i.e., IVW). However, the association was not significantly associated with MR-Egger. Inadequate VC intake was positively associated only with the serum glucose concentrations in the weighted median analysis. The association did not show heterogeneity and horizontal pleiotropy in IVW and MR-Egger tests, but the data distribution was parallel only in IVW analysis. Therefore, the selected SNPs may be insufficient to explain the causal association of VC intake with MetS and hyperglycemia. However, overall results suggest that an inadequate VC intake could significantly contribute to MetS and its components, particularly the serum glucose concentrations. This study is novel, showing the potential causal relationship between the VC intake and MetS and its components, primarily hyperglycemia, in KoGES data, combining Ansan/Ansung plus rural and city hospital-based cohorts. 

VC is an essential nutrient required for collagen synthesis and antioxidant activity, and it has been reported to be associated with various diseases, including scurvy, infection, immunity, cancer, cardiovascular disease, and cognitive function [13,35]. The serum VC concentrations are used to assess a person’s VC status, but they are unsuitable for detecting a latent deficiency or suboptimal status [36]. A meta-analysis with 26 studies showed that the dietary VC from FFQ and dietary recalls is moderately related to the plasma VC concentrations (r = 0.35 for FFQ and r = 0.46 for dietary recall in both genders) [36]. The overall correlation coefficient of both FFQ and dietary recalls is 0.39 in both genders, indicating a moderate correlation. The smoking status in both genders affects the correlation between the dietary and serum VC; the correlation coefficient between the VC intake and serum VC concentration was 0.45 in non-smokers but 0.33 for smokers [11]. It indicates that external factors, such as smoking, increase VC utilization, reducing bioavailability. Therefore, VC intake from diets can be a valuable indicator of VC status. 

VC deficiency is globally related to low- and middle-income countries and low-income groups in high-income countries [37]. Cultural dietary patterns influence VC intake because grain is a staple food and consumes small amounts of other foods, including fruit and vegetables [38]. The present study also showed that income and education were positively associated with the VC intake. The adults in the low VC group consumed mainly rice, and the other food intake was low, which belonged to the rice-main diet pattern. Because Asians have grains as staple foods, vitamins including A, D, B1, and C can be markers of the nutrition status [38]. In KNHANES, the VC intake was positively correlated with a healthy eating index, suggesting that an adequate VC intake can indicate healthy eating to prevent MetS [2]. The present study showed that adults in the low VC group had lower energy intakes than the EER (86.7 %), and they had 1.31 times lower intakes than the high VC group. The low VC group had higher CHO and lowered protein, fat, fiber, calcium, and sodium intake than the high VC group. However, alcohol intake and smokers were lower, and physical activity was higher in the high VC group than in the low VC group. These results suggest that low VC intake could be a potential marker for poor nutrient intake and poor lifestyles. However, other vitamins have not been shown to be associated with type 2 diabetes and MetS. Nevertheless, adults in the low VC group were inversely associated with the incidence of MetS, including waist circumferences, hyperglycemia, dyslipidemia, and hypertension, compared to those in the high VC group. The present study suggested that VC intake can be a potential marker for MetS incidence.

A previous study reported that VC and fruit intake are inversely associated with MetS risk in women, but not in men, in KNHANES, a representative sample of the general South Korean population [12]. A meta-analysis with 28 observational studies showed that VC intake from the diet was inversely associated with MetS by 0.93 times after adjusting for the covariates (*p* = 0.003) [39]. Furthermore, VC concentrations in the circulation had an inverse relationship with MetS risk by 0.60 times in a meta-analysis of 11 observations studies (*p* < 0.001) [39]. Therefore, dietary and serum VC levels may be inversely associated with the MetS risk, suggesting that dietary VC intake may be linked to the serum VC concentration. Zheng et al. reported that genetically predicted plasma VC concentrations showed no strong association with type 2 diabetes in 52,018 adults of European ancestry, even though the plasma VC is inversely associated with type 2 diabetes (hazard ratio and 95% CI = 0.88 (0.82, 0.94)) in the observational study [40]. Furthermore, genetically higher plasma VC concentrations are not significantly associated with cardiovascular events, including coronary artery diseases, ischemic stroke, arterial fibrillation, and heart failure, and their risk factors, such as dyslipidemia, hypertension, and obesity in Europeans [41]. Further study is needed to conduct a causal relationship between serum VC concentration and MetS risk using MR analysis. 

The potential mechanisms of VC deficiency in type 2 diabetes are as follows: (1) VC deficiency elevates ROS content to induce cytotoxicity in various tissues, especially pancreatic β-cells [24]. They are susceptible to ROS, and their cytotoxicity induces type 2 diabetes. Streptozotocin-injected rats induce diabetes by killing pancreatic β-cells and they have lower serum VC concentrations [42]. It suggests that increased oxidative stress elevates pancreatic β-cells, and fewer antioxidants, especially VC, exacerbate their cytotoxicity to develop type 2 diabetes. (2) The transportable form of VC, dehydroascorbic acid, enters the cells through glucose transporters, GLUT1 and GLUT3, in competition with glucose [43]. Hyperglycemia can inhibit dehydroascorbic acid uptake into the cells, which induces VC deficiency. Hyperglycemia causes cellular VC deficiency and increases ROS production, contributing to oxidative stress to make a vicious cycle to type 2 diabetes. In a meta-analysis of short-term clinical studies, VC supplementation improved glycemic control in type 2 diabetic patients [44]. Therefore, VC intake can improve glycemic control, but it needs to be confirmed in a large clinical trial.

No studies have demonstrated the causal relationship of dietary VC intake with MetS and its components by a two-way MR study in Asians. The present study was the first to show a causal relationship between VC intake and MetS in an IVW analysis. Nevertheless, this study had some limitations. First, a liberal statistical level was used (*p* < 5 × 10^−5^) because genetic variants for VC intake did not satisfy the statistical level with a Bonferroni correction (*p* < 5 × 10^−8^). Previous studies showed a liberal statistical level applied to lifestyle instrument variables [27]. Second, the potential residual pleiotropy is challenging to exclude, even though multiple analyses have been conducted to assess pleiotropy [45]. The present study showed that the MR-Egger intercept passed through the origin, indicating no high impact of pleiotropy in the results. Finally, genetic variations are considered a “weak tool” if there is insufficient statistical evidence that genetic variations are related to exposure [46]. Finally, the usual VC intake was estimated from the SQFFQ, validated with the 12-day food records for Korean food intake [47]. However, it could be under-or over-estimated.

## 5. Conclusions

Our results demonstrated that insufficient dietary VC intake could potentially increase the risk of MetS and its metabolic traits, particularly hyperglycemia. In a clinical setting, it should be recommended that adults consume 100 mg/dL VC daily from the diet to improve glycemic control. However, the genetic variants related to VC intake were selected with *p* < 5 × 10^−5^, introducing some bias. Therefore, large-scale, well-designed RCTs on the effect of dietary VC on MetS, particularly type 2 diabetes, will be needed to validate the findings.

## Figures and Tables

**Table 1:** Demographic and biochemical characteristics according to dietary vitamin C (VC) intake status and their association with the VC status.

	Low Intake of VC (*n* = 36,198)	High Intake of VC (*n* = 27,509)	Adjusted OR ^1^ or Beta Coefficients ^2^ and 95% CI
Age (year)	54.6 ± 8.50 ^3^	53.4 ± 8.00 ***	−0.684 (−0.749–−0.618) ^###^
Gender (male, %)	14,183 (39.2) ^4^	9187(33.4) ***	0.563 (0.531–0.597) ^###^
BMI (mg/kg^2^)	24.0 ± 2.94	24.1 ± 2.91 ***	0.485 (0.298–0.672) ^###^
Waist circumferences (cm)	81.5 ± 8.73	81.3 ± 8.71 **	−0.111 (-0.174–−0.049) ^###^
Education (Yes, %)			
≤Middle school High school ≥Collage	15,752 (43.8) 13,227 (36.8) 6954 (19.4)	9661(35.4) *** 11,042 (40.5) 6564 (24.1)	1 1.234 (1.180–1.291) ^###^ 1.466 (1.386–1.550) ^###^
Income (Yes, %)			
≤$2000 $2000–4000 >$4000	12,647 (39.3) 17,436 (54.2) 2069 (6.5)	8142 (32.6) *** 14,728 (59.0) 2087 (8.4)	1 1.136 (1.085–1.189) ^###^ 1.413 (1.287–1.551) ^###^
Smoke (Yes, %)	11,092 (30.7)	7029 (25.6) ***	0.879 (0.828–0.934) ^###^
Alcohol intake (g/day)	4.28 ± 14.3	3.98 ± 12.2 **	−0.021 (-0.061–0.019)
Physical activity (Yes, %)	17,348 (48.0)	15,322 (55.8) ***	1.307 (1.260–1.355) ^###^
Metabolic syndrome (Yes, %)	9855 (27.4)	6960(25.4) ***	0.905(0.873–0.937) ^###^
Serum glucose (mg/dL)	95.7 ± 20.9	94.3 ± 19.2 ***	−0.146 (−0.173–−0.118) ^###^
HbA1c (%)	5.73 ± 0.75	5.72 ± 0.76	−1.014 (−2.062–0.035)
Blood hemoglobin (g/dL)	13.7 ± 0.01	13.9 ± 0.01 **	0.00834 (0.00811–0.00847) ^##^
Hematocrit (%)	41.6 ± 0.02	41.8 ± 0.02 **	0.00423 (0.00394–0.00465) ^#^
Serum total cholesterol (mg/dL)	197 ± 35.8	197 ± 35.5	−0.003 (−0.019–0.012)
Serum HDL (mg/dL)	51.8 ± 13.1	52.3 ± 12.9 ***	0.076 (0.034–0.118) ^###^
Serum LDL (mg/dL)	119 ± 33.0	119 ± 32.8 **	0.010 (−0.006–0.027)
Serum TG (mg/dL)	132 ± 90.8	128 ± 87.5 ***	−0.017 (−0.023–−0.011) ^###^
SBP (mmHg)	123 ± 15.5	122 ± 15.3 ***	−0.166 (−0.201–−0.130) ^###^ −0.048 (−0.102–0.006)
DBP (mmHg)	76.6 ± 10.1	76.4 ± 10.1	
Serum C-reactive protein (mg/L)	0.37 ± 1.57	0.30 ± 1.65 ***	−0.981 (−1.355–−0.607) ^###^
eGFR (mL/min)	83.8 ± 16.4	84.4 ± 16.2 ***	0.127 (0.094–0.161) ^###^
Serum AST (U/L)	24.5 ± 27.4	24.1 ± 16.0	−0.012 (−0.036–0.011) 0.002 (−0.022–0.026)
Serum ALT(U/L)	23.0 ± 25.9	22.9 ± 18.3	

^1^ Adjusted odds ratio (OR) and 95% confidence intervals (CI) represent the association of each categorical variable with dietary VC intake in logistic regression. ^2^ Adjusted beta-coefficient and 95% CI represent the relationship of each continuous variable with dietary VC intake in linear regression. ^3^ Adjusted means ± standard deviations. ^4^ Number of the subjects (percentage of each group). Covariates included age, gender, residence areas, BMI, education, income, daily activity, alcohol intake, and smoking status. ** Significant differences from the low VC intake group at *p* < 0.01 and *** at *p* < 0.001. ^#^ Significant association of the low VC intake group with the designated variable at *p* < 0.05, ^##^ *p* < 0.01 and ^###^ at *p* < 0.001.

**Table 2:** Energy and nutrient intake and dietary inflammation index (DII) according to dietary vitamin C (VC) intake status.

	Low Intake of VC (*n* = 36,198)	High Intake of VC (*n* = 27,509)
Energy (EER%)	86.70 ± 23.47	113.2 ± 35.08 ***
CHO (En%)	73.22 ± 6.67	70.75 ± 7.14 ***
Protein (En%)	12.54 ± 2.24	14.17 ± 2.63 ***
Fat (En%)	12.58 ± 5.31	14.74 ± 5.31 ***
Saturated fat (En%)	6.07 ± 0.028	7.84 ± 0.041 ***
Monounsaturated fat (En%)	8.28 ± 0.031	9.68 ± 0.037 ***
Polyunsaturated fat (En%)	4.43 ± 0.020	5.69 ± 0.023 ***
Cholesterol (mg/day)	126.6 ± 89.77	210.6 ± 147.4 ***
Fiber (g/day)	11.02 ± 4.92	20.97 ± 12.97 ***
Calcium (mg/day)	321.2 ± 154.7	586.3 ± 282.6 ***
Sodium (mg/day)	1890 ± 892	3326 ± 1612 ***
Vitamin C (mg/day)	62.8 ± 22.6	162 ± 72.0 ***
Vitamin B1 (mg/day)	0.814 ± 0.297	1.255 ± 0.519 ***
Vitamin A (μg/day)	312.1 ± 154.4	678.8 ± 412.1 ***
Vitamin D (ug/day)	5.70 ± 0.29	7.28 ± 0.33 **
DII (scores)	−14.49 ± 0.08	−26.85 ± 0.09 ***

The values represent means ± standard deviations. EER, estimated energy requirement; CHO, carbohydrate; En%, percentage of energy intake. ** Significant differences from the low VC intake group at *p* < 0.01 and *** at *p* < 0.001.

**Table 3:** Association of dietary vitamin C intake with metabolic syndrome and its metabolic traits using genetic variant randomization using two-sample Mendelian randomization (MR).

	MR			Heterogeneity			Pleiotropy	
Exposures	Method	OR (95% CI) ^1^	*p*-value	Q	*p*-value	Intercept	SE	*p*-value
Metabolic syndrome	MR Egger	1.615 (0.639–4.086)	0.317	2.724	1	−0.006	0.030	0.855
	WMD	1.295 (0.815–2.057)	0.274					
	IVW	1.491 (1.034–2.150)	0.032	2.758	1			
	WMO	1.196 (0.483–2.962)	0.700					
Hypertension	MR Egger	1.490 (0.623–3.563)	0.375	2.973	1	0.003	0.028	0.924
	WMD	1.423 (0.907–2.234)	0.125					
	IVW	1.550 (1.099–2.185)	0.012	2.982	1			
	WMO	1.390 (0.560–3.454)	0.482					
Exposures	Method	β (95% CI)	*p*-value	Q	*p*-value	Intercept	SE	*p*-value
Waist circumferences (cm)	MR Egger	0.558 (−0.564–1.679)	0.336	3.728	1	−0.0007	0.036	0.985
	WMD	0.402 (−0.153–0.958)	0.156					
	IVW	0.548 (0.106–0.989)	0.015	3.728	1			
	WMO	0.282 (−0.851–1.416)	0.628					
Serum glucose concentrations (mg/dL)	MR Egger	0.773 (−0.657–2.204)	0.296	1.930	1	−0.007	0.046	0.883
	WMD	0.755 (0.055–1.455)	0.035					
	IVW	0.674 (0.113–1.235)	0.018	1.952	1			
	WMO	0.857 (−0.451–2.164)	0.206					
Serum triglyceride concentrations (mg/dL)	MR Egger	0.713 (−0.345–1.772)	0.194	6.852	1	−0.016	0.034	0.651
	WMD	0.187 (−0.351–0.725)	0.495					
	IVW	0.487 (0.072–0.902)	0.021	7.059	1			
	WMO	0.133 (−0.937–1.202)	0.809					
Serum HDL concentrations (mg/dL)	MR Egger	0.349 (−0.510–1.207)	0.431	2.827	1	0.005	0.028	0.862
	WMD	0.352 (−0.092–0.797)	0.120					
	IVW	0.419 (0.081–0.758)	0.015	2.858	1			
	WMO	0.172 (−0.735–1.080)	0.712					

^1^ Reference was the dietary high VC intake (≥100 mg/dL) in logistic regression. WMD, weighted median; IVW, inverse-variance weighting; WMO, weighted mode; SE, standard error.

## Data Availability

Data is contained within the article and supplementary material.

## Supplementary Materials

The following supporting information can be downloaded at: https://www.mdpi.com/article/10.3390/antiox11050857/s1, Table S1. Genetic variants and effect alleles with frequencies and their magnitude on VC intake and their associations with serum triglyceride concentrations. Figure S1. Leave-one-out sensitivity analysis of MR for the dietary VC intake on metabolic syndrome and its metabolic traits.
