# Supplementary material for: A Causal Relationship between Vitamin C Intake with Hyperglycemia and Metabolic Syndrome Risk: A Two-Sample Mendelian Randomization Study
Source: Antioxidants (Basel). 2022 Apr 27;11(5):857. doi: 10.3390/antiox11050857 (PMC9137888; doi:10.3390/antiox11050857)
Supplement: Supplementary file 1 [file antioxidants-11-00857-s001.zip › antioxidants-1672362-supplementary.pdf]

Supplemental Table S1. Genetic variants associated with V-C intake in city cohort and their magnitude in city hospital-based cohort and their association with fasting serum glucose concentrations at a fasting state in Ansan/Ansung+rural cohort

CHR	SNP	EA	NA	Beta for V-C intake	SE for V-C intake	p-value	Beta for fasting serum glucose	SE for fasting serum glucose	*p*-value	Gene name
1	rs74682528	C	T	0.0569	0.0320	4.19E-05	0.0212	0.1095	0.6554	KAZN
1	rs778897343	G	C	-0.0404	0.0223	2.93E-05	0.0048	0.0764	0.8902	NME7
1	rs3737682	A	G	-0.0565	0.0292	8.06E-06	-0.0559	0.1087	0.2361	SLC19A2
3	rs34304469	G	C	-0.1004	0.0557	3.32E-05	0.0846	0.1885	0.3008	FANCD2
3	rs141979010	T	C	-0.1047	0.0572	2.49E-05	-0.0931	0.2123	0.3124	ACP3
3	rs3792330	C	G	-0.1003	0.0567	4.55E-05	-0.1013	0.212	0.2711	ACP3
3	rs1410094297	G	A	0.0934	0.0502	1.83E-05	-0.1201	0.1934	0.1526	ENSG00000240478
4	rs138657647	G	A	0.0952	0.0494	9.59E-06	0.1007	0.1523	0.1274	ANK2
4	rs147057943	G	C	0.0803	0.0429	1.71E-05	-0.0584	0.15	0.3697	ARHGAP10
4	rs148412038	G	T	-0.1085	0.0553	6.28E-06	0.0204	0.1958	0.8117	FSTL5
5	rs79856437	G	T	-0.0534	0.0290	2.19E-05	0.0346	0.0978	0.4154	ENSG00000248752
5	rs79782138	A	G	-0.0488	0.0274	4.07E-05	-0.0489	0.0987	0.2541	GRAMD2B
5	rs144093322	A	G	0.0630	0.0322	6.71E-06	-0.0065	0.1106	0.8928	LINC01184
5	rs1335330583	G	A	0.0438	0.0239	2.54E-05	-0.0438	0.0830	0.2238	LOC102724404
7	rs185093326	C	T	-0.0925	0.0510	2.94E-05	-0.0083	0.1753	0.9127	GRID2IP
7	rs189780376	A	T	-0.1076	0.0582	2.05E-05	-0.036	0.2148	0.7007	Unknown
7	rs187373518	A	G	-0.1090	0.0581	1.56E-05	-0.0364	0.2147	0.6967	Unknown
7	rs185583852	C	T	-0.1090	0.0581	1.56E-05	-0.0364	0.2147	0.6967	Unknown
9	rs75760232	A	G	-0.1228	0.0655	1.56E-05	-0.2047	0.2703	0.0811	Unknown
9	rs140834132	G	C	-0.1344	0.0638	1.22E-06	-0.1967	0.262	0.0838	Unknown
9	rs199788138	C	T	0.1261	0.0676	1.75E-05	-0.0534	0.2344	0.5998	HACD4
10	rs1232162456	C	T	0.0580	0.0328	4.42E-05	-0.0754	0.1135	0.126	KCNMA1
10	rs138051726	G	A	0.1106	0.0625	4.53E-05	0.0107	0.2018	0.9037	CYP2C60P
10	rs1419006605	CA	C	-0.0490	0.0268	2.55E-05	0.0082	0.0920	0.8358	TDRD1
11	rs149974677	T	A	0.0878	0.0487	3.35E-05	0.0671	0.158	0.328	Unknown
12	rs3751299	A	G	0.0398	0.0222	3.89E-05	-0.0316	0.0753	0.3338	FBRSL1
14	rs1397604872	G	C	-0.1229	0.0691	4.16E-05	0.1222	0.2097	0.1795	EIF5
15	rs117035029	A	G	0.0959	0.0492	6.98E-06	0.0803	0.1598	0.2484	SORD
15	rs76071295	A	G	0.0959	0.0492	6.98E-06	0.0803	0.1598	0.2484	SORD
15	rs118153043	C	T	0.0959	0.0492	6.98E-06	0.0803	0.1598	0.2484	SORD
15	rs139808555	G	T	0.0959	0.0492	6.98E-06	0.0803	0.1598	0.2484	SORD
16	rs1166633603	C	A	0.0678	0.0376	3.28E-05	0.0871	0.1192	0.0932	CDH13
17	rs16963856	A	G	0.0438	0.0235	1.77E-05	-0.0123	0.0806	0.7246	LOC105371740
17	rs231475	G	A	0.0430	0.0235	2.41E-05	0.0354	0.0773	0.2884	MPP2
17	rs75665319	C	T	0.0523	0.0264	4.55E-06	-0.0253	0.0902	0.5181	SMURF2,
17	rs74251270	T	C	0.0527	0.0266	5.41E-06	-0.0177	0.0903	0.6512	SMURF2
18	rs1206393585	T	C	0.0414	0.0204	2.73E-06	0.0082	0.0696	0.7887	LAMA1
18	rs1341776932	C	CT	-0.0302	0.0163	2.05E-05	-0.0283	0.0565	0.2491	MIR4527HG
19	rs148415596	G	C	-0.0775	0.0430	3.29E-05	-0.052	0.1586	0.4487	CLASRP

19	rs191480708	T	C	-0.0808	0.0427	1.29E-05	-0.0479	0.1571	0.4828	CLASRP
22	rs192599903	C	T	0.1326	0.0727	2.62E-05	0.0026	0.2393	0.9803	TTC28
22	rs116951565	G	A	-0.0856	0.0481	4.14E-05	-0.0727	0.1848	0.3649	BPIFC

CHR, chromosome; EA, effective allele; NA, non-effective allele; SE, standard errors

A

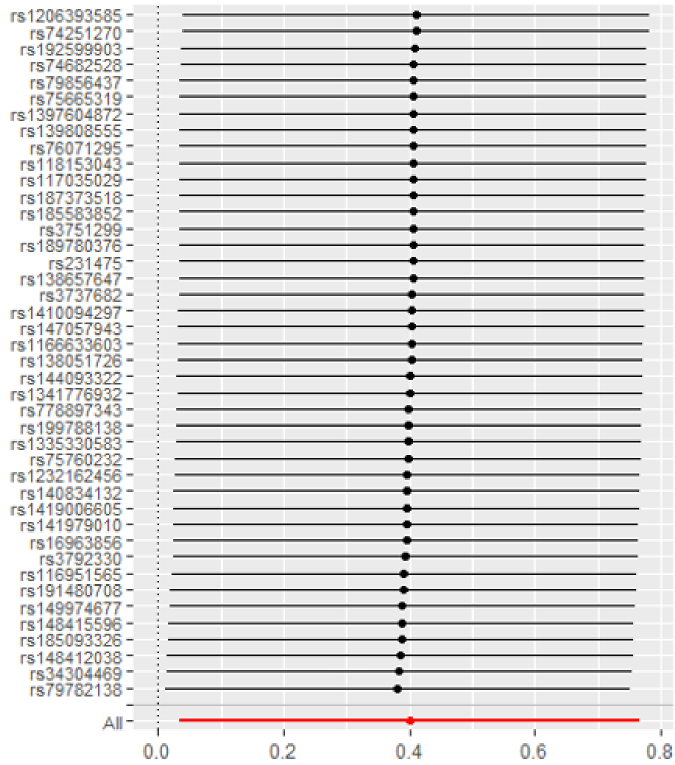

MR leave-one-out sensitivity analysis for ‘Vitamin C intake’ on ‘Metabolic syndrome’

B

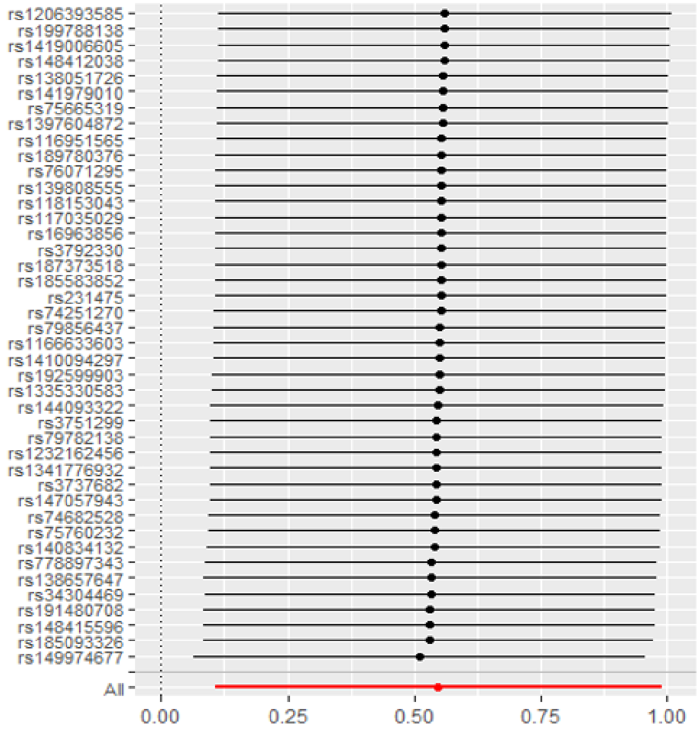

MR leave-one-out sensitivity analysis for ‘Vitamin C intake’ on ‘Waist circumferences’

C

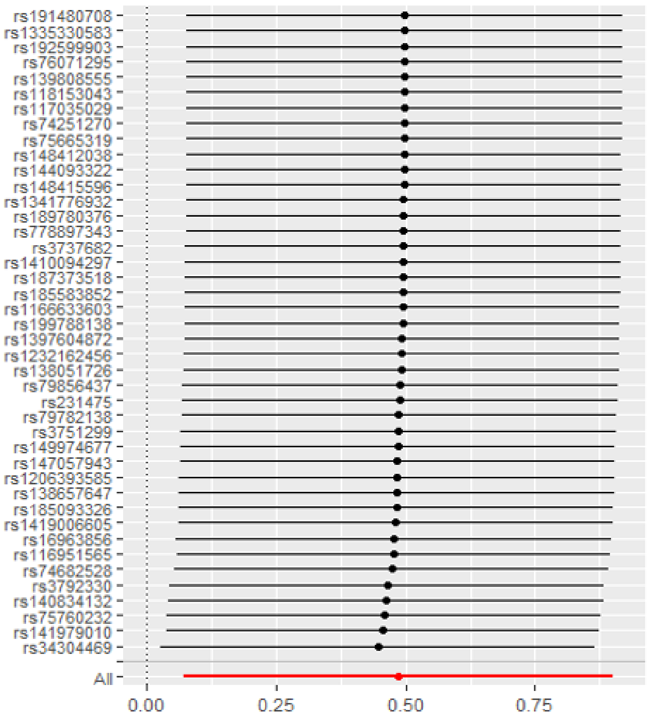

MR leave-one-out sensitivity analysis for 'Vitamin C intake' on 'Serum triglyceride concentrations'

D

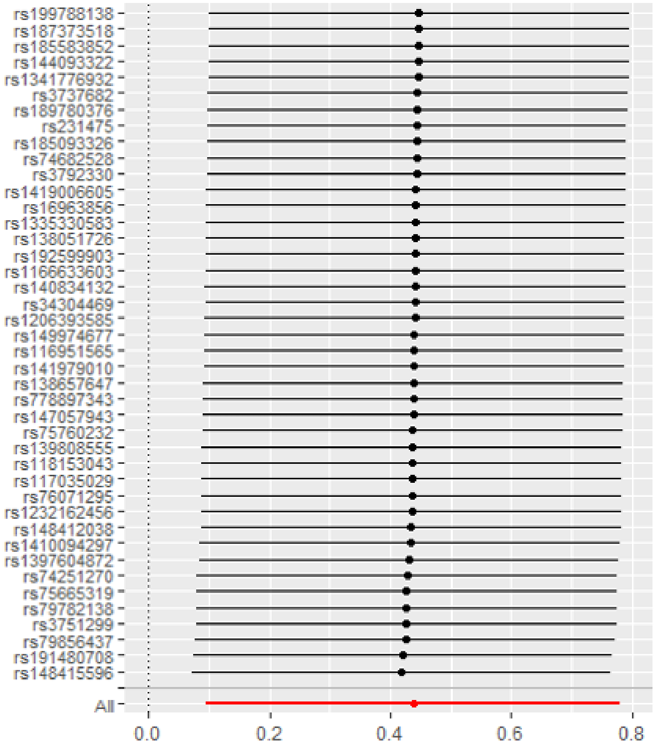

MR leave-one-out sensitivity analysis for 'Vitamin C intake' on 'Hypertension'

E

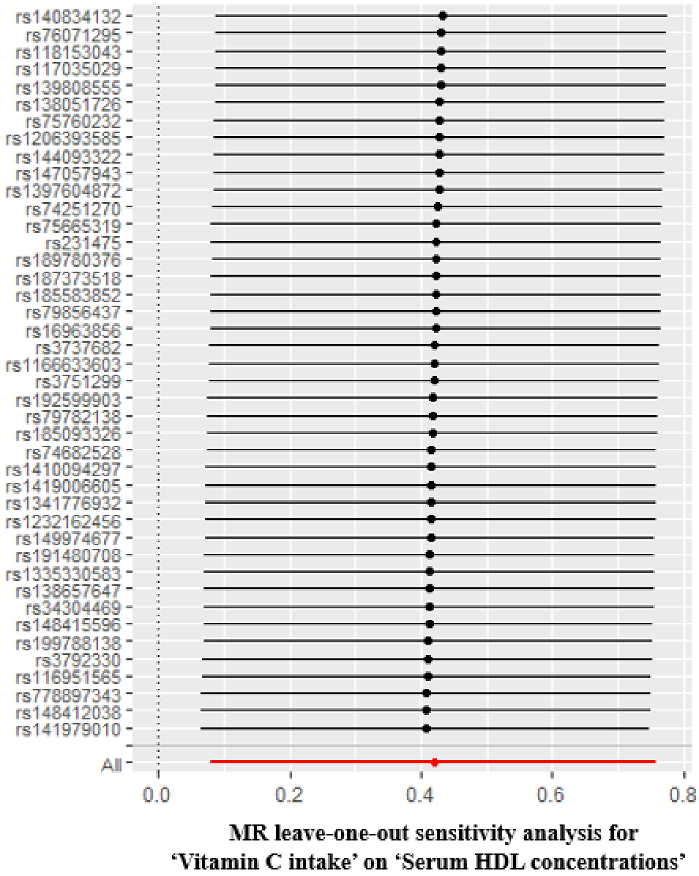

Supplemental Figure S1. Leave-one-out sensitivity analysis of MR for the dietary V–C intake on metabolic syndrome and its metabolic traits. A. Metabolic syndrome, B. Waist circumferences, C. serum triglyceride concentrations, D. Hypertension, E. Serum HDL concentrations
